# Tumor suppressor BAP1 emerges as a key regulator of metabolism and cell death in tumors

**DOI:** 10.3389/fcell.2026.1868659

**Published:** 2026-07-10

**Authors:** Kexin Fan, Jun Yao, Shaobo Wu, Yilei Zhang

**Affiliations:** 1 Department of Biochemistry and Molecular Biology, School of Basic Medical Sciences, Xi’an Jiaotong University, Xi’an, Shaanxi, China; 2 Henan Key Laboratory of Cancer Epigenetics, Cancer Institute, The First Affiliated Hospital, College of Clinical Medicine of Henan University of Science and Technology, Luoyang, Henan, China

**Keywords:** BAP1, cell death, deubiquitinase, epigenetic, metabolism, tumors

## Abstract

BRCA1-associated protein 1 (BAP1) is a deubiquitinase (DUB) localized in both the nucleus and cytoplasm and is widely recognized as a tumor suppressor. Germline and somatic alterations in BAP1 have been strongly associated with increased susceptibility to diverse cancer types and with adverse clinical outcomes. Although BAP1 is best known for its role in epigenetic regulation, particularly through the modulation of histone H2A monoubiquitination (H2Aub) and gene transcription, accumulating evidence suggests that its functional repertoire extends well beyond these canonical activities. BAP1 is increasingly viewed as a central molecular hub through which fundamental cellular processes are integrated and coordinated *via* its catalytic activity and dynamic protein interaction networks. In this review, the pleiotropic functions of BAP1 are systematically examined across several biological dimensions, including epigenetic regulation, genomic stability, cellular metabolism, and cell fate determination. Notably, BAP1-mediated regulation is highly context dependent, as cell type, differentiation status and tumor microenvironmental (TME) cues may shape its downstream effects and contribute to heterogeneous biological outcomes. By synthesizing these multidimensional regulatory mechanisms, this review provides an integrated overview of the molecular features and functional roles of BAP1, with particular emphasis on its impacts on the regulation of cell death including apoptosis, ferroptosis and disulfidptosis. Collectively, these insights underscore the evolving understanding of BAP1 biology over the past decade and highlight the need for renewed attention to this critical tumor suppressor and its therapeutic potential in cancer.

## Introduction

Cancer represents a major global public health burden, and its initiation and progression are driven by a multifaceted interplay among genetic alterations, epigenetic dysregulation, metabolic reprogramming, and evasion of regulated cell death. In this context, functional inactivation of tumor suppressor genes is considered a pivotal event in malignant transformation (Sherr, 2004). Since its initial identification as a BRCA1-binding partner, BRCA1-associated protein 1 (BAP1) has been widely recognized as a critical tumor suppressor (Jensen et al., 1998). BAP1 encodes a deubiquitinase (DUB) that functions in both the nucleus and cytoplasm and is involved in the regulation of chromatin-associated events, including histone H2A monoubiquitination (H2Aub) and transcriptional control, as well as broader cellular processes such as DNA damage responses, metabolism, and cell fate regulation. Germline mutations in BAP1 cause BAP1 tumor predisposition syndrome (BAP1-TPDS), an autosomal dominant disorder characterized by a markedly increased lifetime risk of malignancies, including malignant mesothelioma, uveal melanoma, and renal cell carcinoma (Walpole et al., 2018). Additionally, somatic BAP1 mutations are frequently detected in diverse sporadic cancers and are often associated with more aggressive disease courses and adverse clinical outcomes (Carbone et al., 2020).

Accumulating evidence has substantially broadened our understanding of the functional scope of BAP1. BAP1 is now viewed not as a single purpose factor but as a dynamic molecular hub that interacts with diverse protein partners, through which several core cellular processes are integrated and modulated, including DNA damage repair, cell cycle control, chromatin modification, programmed cell death, and the immune response (Wang et al., 2016). This multilayered regulatory capacity positions BAP1 as a central regulator of cellular homeostasis. Notably, in certain cellular contexts or tumor microenvironmental settings, BAP1 or its associated complexes may exert context-specific, noncanonical functions, such as stabilizing selected oncoproteins or enhancing therapeutic resistance, which may appear to contradict its established tumor-suppressive role (Liu et al., 2018; Zhang et al., 2023). These observations underscore the highly context-dependent nature of BAP1 activity and its complex involvement in cancer biology.

### Overview of the molecular structure and biological functions of BAP1

The diverse functions of BAP1 including its essential role as a molecular hub that coordinates epigenetic regulation, genomic stability, and metabolism, are underpinned by its distinct structural domains. These structural domains provide specialized modules for catalysis, protein interactions, and subcellular targeting, thereby establishing the framework through which a broad range of downstream biological processes is regulated by BAP1.

### Molecular basis of BAP1

**FIGURE 1:**
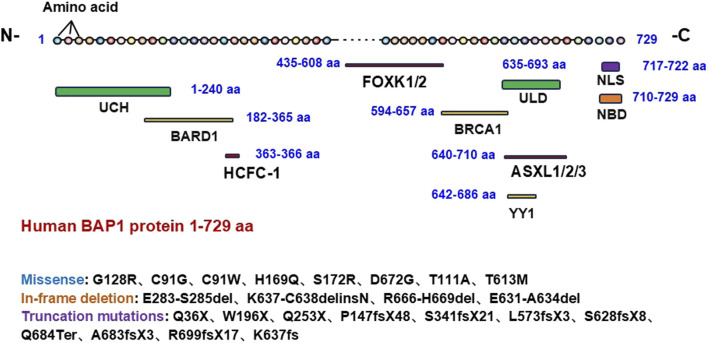
Domain architecture of human BAP1 and the locations of BAP1 mutations. The figure shows the domain architecture of human BAP1 and representative mutations and deletions identified in different cancer types. Major functional domains highlighted include the UCH domain, ULD, regions required for protein-protein interactions, NBD, and NLS. The figure also annotates representative BAP1 genetic alterations identified in malignancies such as uveal melanoma, mesothelioma and others. UCH: ubiquitin C-terminal hydrolase; ULD: UCH37-like domain; NBD: nucleosome-binding domain; NLS: nuclear localization signals.

BAP1 was initially identified in 1998 during functional studies of BRCA1 and was named on the basis of its direct interaction with the BRCA1 RING domain. It encodes a 729-amino-acid deubiquitinase (Figure 1). Its key catalytic domain, the ubiquitin C-terminal hydrolase (UCH) domain, contains the C91-H169-D184 catalytic triad, which is responsible for hydrolyzing the isopeptide bond between ubiquitin and its substrate (Jensen et al., 1998; Ge et al., 2023).

The structure and functions of BAP1 have been highly conserved throughout evolution. In *Drosophila*, the BAP1 homolog Calypso forms the polycomb repressive deubiquitinase (PR-DUB) complex with the ASX protein, specifically removing histone H2AK118ub (the monoubiquitination of histone H2A at lysine 118) modifications (Scheuermann et al., 2010). In human cells, BAP1 is characterized by a modular architecture. In addition to its N-terminal catalytic domain, the C terminus contains a proline-tyrosine nuclear localization signal (PY-NLS), which mediates nuclear import, and a nucleosome-binding domain (NBD) (Yang et al., 2022). An extended intermediate region of approximately 395 amino acids functions as a linker and stabilizing element and also provides a platform for protein-protein interactions (Sahtoe et al., 2016; Yang et al., 2022). BAP1 interacts with multiple proteins, including BRCA1, BARD1, ASXL1/2/3, FOXK1/2, YY1, HCFC1, OGT, and KDM1B, thereby forming an extensive regulatory network (Jensen et al., 1998; Machida et al., 2009; Nishikawa et al., 2009; Yu et al., 2010; Carbone et al., 2013; Okino et al., 2015; Sahtoe et al., 2016; Campagne et al., 2019). Mutations in these critical domains directly underlie the functional diversity of BAP1 alterations. Domain impairment can disrupt interactions with specific partner proteins and perturb downstream biological processes. This domain-dependent disruption may partly explain the complexity of tumor phenotypes associated with BAP1 mutations (Harbour et al., 2010; Eletr and Wilkinson, 2011; Okino et al., 2015; Peng et al., 2018).

A central function of BAP1 is its role as the catalytic subunit of the PR-DUB complex, through which monoubiquitination of histone H2A at lysine 119 (H2AK119ub) is reversed. H2AK119ub is a chromatin-associated epigenetic modification catalyzed by polycomb repressive complex 1 (PRC1) and is commonly associated with transcriptional repression, thereby contributing to transcriptional regulation (Tamburri et al., 2022). In 2023, Xu et al. elucidated that nucleosome anchoring by BAP1 is mediated by a positively charged “finger” region within its C-terminal extension and a highly conserved arginine-rich “RRSRR” motif in the UCH domain. This structural arrangement enables the precise delivery of the H2AK119ub tail to the catalytic site for deubiquitination (Ge et al., 2023). These studies not only resolved the high-resolution cryo-EM structure of the histone H2A deubiquitinase bound to ubiquitinated nucleosomes but also revealed the molecular mechanism underlying the selective removal of H2AK119ub1 by the PR-DUB complex. These findings substantially advance our understanding of histone H2A deubiquitination and provide a structural basis for the rational development of therapeutics targeting BAP1 and ASXL1.

### Regulation of genomic stability

Substantial evidence supports the role of BAP1 as a critical guardian of genomic stability, primarily through its regulation of DNA damage repair and replication fidelity (Jeggo et al., 2016; Kwon et al., 2023). These findings provide important insights into the pathogenesis of BAP1-associated cancers and the development of targeted therapeutic strategies (Carbone et al., 2020; Yue et al., 2023). Within the homologous recombination pathway, BAP1 is specifically recruited to DNA double-strand breaks, where its deubiquitinase activity promotes the accumulation of key repair factors, including RAD51 and BRCA1, thereby enhancing repair efficiency (Ulrich and Walden, 2010; Yu et al., 2014). Loss of BAP1 renders cells highly sensitive to ionizing radiation and PARP inhibitors, resembling the synthetic lethality observed in BRCA-deficient tumors and highlighting BAP1 as a potential therapeutic target (Bryant et al., 2005; Farmer et al., 2005; Toma et al., 2019). This repair function is further regulated by phosphorylation at residues such as Ser592, independently of its deubiquitinase activity (Ismail et al., 2014; Yu et al., 2014). BAP1 is also required for efficient nucleotide excision repair. Its deficiency impairs repair capacity and increases cellular sensitivity to ultraviolet radiation (Lee et al., 2022). Recruitment of BAP1 to sites of DNA damage depends on poly (ADP-ribose) polymerase 1 (PARP1) activity and H2A ubiquitination marks, whereas PARylation at glutamate 31 is essential for maintaining protein stability and DNA repair capacity, mutations affecting this residue have been linked to cancer (Ismail et al., 2014; Lee et al., 2022). Furthermore, BAP1 contributes to DNA replication fidelity through stabilization of the INO80 chromatin-remodeling complex and plays a crucial role in maintaining chromosomal stability. It ensures accurate chromosome segregation by stabilizing key mitotic factors such as γ-tubulin and DIDO1 (Lee et al., 2014; Zarrizi et al., 2014; Xiao et al., 2020). BAP1 deficiency leads to chromosomal aberrations, increased aneuploidy, and even complex genomic rearrangements (Shen, 2013; Lee et al., 2014; Yu et al., 2014; Mansfield et al., 2019; Oey et al., 2019; Perkail et al., 2020). Through its coordinated regulation of DNA damage repair, replication fidelity, and chromosome segregation, BAP1 serves as a central guardian of genomic stability.

### Regulation of gene transcription and expression

BAP1 is not a conventional transcription factor but instead functions primarily as a transcriptional coregulator. In mammalian cells, BAP1 primarily functions as a transcriptional coactivator. Through the PR-DUB complex, it removes the repressive histone mark H2AK119ub, thereby promoting gene expression (Campagne et al., 2019; Fursova et al., 2021). In mesothelioma associated with BAP1 cancer syndrome, loss of BAP1 function leads to aberrant accumulation of H2AK119ub, chromatin compaction, and silencing of tumor suppressor genes, ultimately facilitating tumorigenesis (Galateau-Sallé, 2025). BAP1-mediated epigenetic regulation frequently cooperates with other activating chromatin-modifying mechanisms. BAP1 forms complexes with histone modifiers such as KMT2C and KDM6A at enhancer regions, thereby promoting the deposition of activating marks, including H3K27ac and H3K4me1/me3, promoting chromatin accessibility, and ultimately enhancing transcription of downstream target genes (Yu et al., 2010; Wang et al., 2018; Campagne et al., 2019; He et al., 2019; Kolovos et al., 2020). In addition, interactions with regulatory partners such as ASXL proteins, FOXK1/2, and HCFC1 direct BAP1 activity toward specific genomic loci and biological pathways.

Of note, the transcriptional regulatory function of BAP1 is highly context dependent and exhibits a dual nature, allowing it to mediate transcriptional repression under specific biological conditions. For example, BAP1 can silence genes such as γ-globin through the deubiquitination and stabilization of the transcriptional corepressor NCOR1 (Yu et al., 2018). It can also repress SLC7A11 expression by reducing H2A ubiquitination at its promoter, thereby sensitizing cells to ferroptosis (Zhang et al., 2018). Studies in embryonic stem cells further highlight the complexity of BAP1-mediated regulation. BAP1 is essential for maintaining the appropriate genomic distribution of H2AK119ub and H3K27me3, and its loss results in widespread redistribution of these repressive histone marks. Therefore, global chromatin compaction and transcriptional suppression occur alongside selective derepression of specific polycomb target genes, ultimately disrupting transcriptional homeostasis (Conway et al., 2021; Fursova et al., 2021).

The transcriptional output of BAP1 is highly context dependent and cannot be simply classified as uniformly activating or repressive. A useful framework is to consider BAP1 as a chromatin context-dependent transcriptional coregulator whose functional direction is determined by local chromatin state, recruitment factors, stress conditions, subcellular localization, and the integrity of its PR-DUB complex. At transcriptionally active genes or enhancers, BAP1 generally acts as a co-activator by removing H2AK119ub or modulating chromatin state. In contrast, BAP1 can also support transcriptional repression under defined biological contexts. One example is the repression of SLC7A11. Interestingly, BAP1 and PRC1 have opposite effects on promoter H2Aub but can both contribute to SLC7A11 repression, suggesting that dynamic H2Aub turnover, rather than absolute H2Aub abundance alone, determines transcriptional output at this locus (Zhang et al., 2019). Another example occurs under glucose deprivation, where BAP1 binds to the promoters of ATF3 and CHOP and represses the metabolic stress-induced unfolded protein response transcriptional network (Dai et al., 2017). In this setting, BAP1 suppresses unresolved endoplasmic reticulum (ER) stress, reactive oxygen species (ROS) accumulation, ATP depletion, and stress-induced apoptosis. BAP1 can also repress gene expression indirectly by stabilizing corepressor machinery such as NCOR1 (Yu et al., 2018). The cellular types and activity of BAP1-interacting factors further influence BAP1 functions. O-GlcNAcylation of FOXK1, which is linked to glucose availability and peaks around the G1/S transition, enhances FOXK1–BAP1 interaction and promotes BAP1 recruitment to E2F target genes, thereby activating cell-cycle transcriptional programs (Ahmed et al., 2025). Under therapeutic pressure, such as sustained CDK4/6 inhibition in hepatobiliary cancers, BAP1 can remove H2AK119ub from the TCF4 promoter and activate TCF4/WNT/EMT programs, supporting tumor cell plasticity and drug resistance (Feng et al., 2026). Although current evidence suggests that subcellular localization does not directly determine the transcriptional directionality of BAP1, proper nuclear localization remains essential for its transcriptional regulatory functions (Yu et al., 2018). By actively or passively integrating upstream signals and executing epigenetic reprogramming as well as downstream regulatory functions, BAP1 plays a complex yet central role in transcriptional regulation.

### Biomolecular modification and protein homeostasis

As a deubiquitinase, BAP1 mediates key epigenetic modifications and directly or indirectly regulates the ubiquitination status of multiple genes and proteins, thereby influencing gene expression and protein homeostasis. BAP1 has been shown to stabilize multiple substrate proteins through deubiquitination, including IRF3, MYCN, LKB1, MAFF, HIF-1α, and LATS2, thereby broadly contributing to immune responses, tumorigenesis, energy metabolism, and signal transduction (Brekken, 2020; Yang et al., 2020; Bononi et al., 2023; Zhang et al., 2023; Liu et al., 2024; Xie et al., 2024). In parallel, BAP1 function is dynamically regulated by its intrinsic self-deubiquitination activity and diverse post-translational modifications. Its nuclear localization and activity depend on a self-regulatory circuit. The ubiquitin-conjugating enzyme UBE2O catalyzes non-degradative ubiquitination within the BAP1 nuclear localization signal region, resulting in cytoplasmic sequestration. This modification can be reversed by wild-type BAP1 through its intrinsic enzymatic activity. The nuclear transport protein TNPO1 recognizes the BAP1 C-terminal PY-NLS motif. TNPO1 not only facilitates BAP1 nuclear import but also directly antagonizes UBE2O, thereby ensuring proper BAP1 nuclear localization and tumor-suppressive activity. Disruption of this circuit represents an important mechanism underlying the functional inactivation of certain cancer-associated BAP1 mutants (Mashtalir et al., 2014; Yang et al., 2022).

Post-translational modifications of BAP1 provide an additional regulatory layer that links upstream stress signals to tumor-suppressive or context-dependent outputs. Among these modifications, PARP1-mediated PARylation is particularly relevant to DNA damage-associated tumorigenesis. Upon UV-induced DNA damage, PARP1 interacts with BAP1 and promotes its recruitment to damaged chromatin, a process that also depends on H2AK119ub at damage sites. PARP1 can transiently PARylate BAP1 and regulate its enzymatic activity, protein stability, and damage-site recruitment. Several BAP1 PARylation sites are mutated in human cancers; notably, Glu31, which is recurrently altered in kidney cancer, is required for BAP1 stabilization, UV-induced DNA damage repair, and suppression of kidney cancer cell viability. These findings connect BAP1 PARylation to nucleotide excision repair, genome stability, and gene-environment interactions in cancer (Lee et al., 2022).

Phosphorylation regulates BAP1 through a different but complementary mechanism. DNA damage-induced phosphorylation, including phosphorylation at Ser592 and ATM-dependent phosphorylation, links BAP1 to the DNA damage response and double-strand break repair. In this context, phosphorylation does not merely alter basal BAP1 abundance, but also helps coordinate the recruitment and function of BAP1-containing PR-DUB complexes at damaged chromatin, thereby contributing to homologous recombination, recovery from DNA damage, and genome stability (Eletr et al., 2013). More recently, AMPK-mediated phosphorylation has connected BAP1 to metabolic stress and tumor suppression. Under glucose starvation, 2-deoxyglucose treatment, or metformin exposure, AMPKα phosphorylates BAP1 at S123, S469, and S583, strengthening the BAP1-pVHL interaction and promoting pVHL deubiquitination and stabilization. Either AMPKα depletion or phosphorylation-deficient BAP1 disrupts the above phosphorylation, destabilizing pVHL and driving tumor progression in cell and patient-derived xenograft models (Li et al., 2026).

Thus, PARylation and phosphorylation appear to control different dimensions of BAP1 biology. PARylation primarily couples BAP1 to PARP1-dependent DNA damage recognition, nucleotide excision repair, enzymatic regulation, and protein stability, whereas phosphorylation translates DNA damage or metabolic stress signals into BAP1-dependent repair or tumor-suppressive programs. Other modifications further diversify BAP1 regulation. UFMylation has been reported to activate BAP1 and promote pVHL stabilization, thereby suppressing colorectal cancer progression (Yang et al., 2025), whereas glutamylation/deglutamylation modulates BAP1 stability and hematopoietic stem cell self-renewal (Xiong et al., 2020). These findings suggest that disease-associated BAP1 dysfunction can arise not only from genetic mutation or deletion but also from disruption of stress-responsive post-translational regulatory circuits.

### Metabolic regulation by BAP1

**FIGURE 2:**
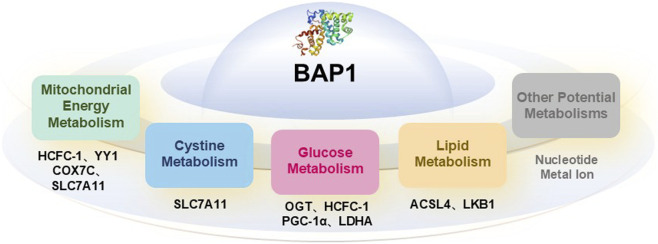
Schematic illustration of the proposed regulatory roles of BAP1 in cellular metabolic pathways. BAP1 is involved in the regulation of mitochondrial energy metabolism, cystine metabolism, glucose metabolism, lipid metabolism, and other potential metabolic processes, including nucleotide metabolism and metal ion metabolism through its associated targets. The diagram summarizes the currently proposed links between BAP1 and metabolic regulation based on available evidence.

Dynamic alterations in cellular metabolism are key drivers of the pathogenesis and progression of numerous diseases, including inflammation, diabetes, and cancer, which are closely associated with metabolic dysregulation (Goodpaster and Sparks, 2017; Singh et al., 2019). BAP1 has also been implicated in the multifaceted regulation of cellular metabolism and modulates several key metabolic pathways through complexes formed with its interacting proteins (Han et al., 2021) (Figure 2).

#### Mitochondrial energy metabolism

BAP1 has been established as essential for the maintenance of normal mitochondrial function. BAP1 depletion induces dysregulation of multiple metabolism-related genes, among which mitochondrial function–related genes are particularly affected. The transcription factor YY1 specifically recruits the BAP1/HCFC-1 complex to regulate COX7C expression, which encodes a component of mitochondrial electron transport chain complex IV. This process is crucial for maintaining mitochondrial oxidative phosphorylation and energy production (Yu et al., 2010). Furthermore, aberrant BAP1 function may shift cellular metabolic programs toward cancer-associated phenotypes, suggesting a potential link between BAP1-related mitochondrial dysfunction and cancer development (Bononi et al., 2017b). Rohatgi et al. demonstrated that the BAP1 deubiquitinase activity induces metabolic reprogramming in osteoclasts by reducing ROS levels and impairing mitochondrial energy metabolism. In BAP1-deficient osteoclasts, SLC7A11 is hyperactivated, resulting in excessive intracellular glutamate consumption and limiting its entry into the tricarboxylic acid (TCA) cycle. These changes result in reduced levels of TCA cycle intermediates (e.g., succinate, malate), impaired mitochondrial respiration and ATP synthesis, and widespread downregulation of genes associated with amino acid metabolism (Rohatgi et al., 2023).

#### Cystine metabolism

Through its deubiquitinase activity, the tumor suppressor BAP1 directly modulates a central checkpoint in cystine metabolism at the epigenetic level. Specifically, BAP1 represses transcription of the cystine transporter SLC7A11 by decreasing H2Aub at the SLC7A11 promoter, thereby limiting extracellular cystine uptake (Zhang et al., 2018). Once inside the cell, cystine is rapidly reduced to cysteine, the rate-limiting precursor for synthesis of the major antioxidant glutathione (GSH). By restricting GSH production, BAP1 attenuates cellular antioxidant defenses and markedly sensitizes cells to ferroptosis. Conversely, loss of BAP1 function through mutation or deletion upregulates SLC7A11 expression, driving excessive cystine import and GSH synthesis. BAP1-mediated repression of SLC7A11 occurs independently of canonical stress transducers such as NRF2 and ATF4 (Ye et al., 2014). Notably, BAP1 functions synergistically with and counterbalances PRC1, which catalyzes H2A ubiquitination. Together, BAP1 and PRC1 maintain a dynamic equilibrium of H2A ubiquitination at the SLC7A11 promoter, rather than simply controlling its absolute level, to suppress SLC7A11 transcription and regulate cystine metabolic homeostasis (Zhang et al., 2019). This epigenetic regulatory pathway underscores the central role of BAP1 in cellular cystine metabolism.

#### Glucose metabolism

BAP1 contributes to the maintenance of glucose homeostasis through complex molecular mechanisms. Through cooperation with the OGT/HCFC-1 complex, BAP1 contributes to O-GlcNAcylation-dependent stabilization of PGC-1α, a key regulator of gluconeogenesis, thereby preventing its degradation through the ubiquitin–proteasome pathway. This modification not only alters the molecular conformation and functional state of PGC-1α but also facilitates BAP1 recruitment. Once recruited to PGC-1α, BAP1 further stabilizes this protein and thereby facilitates efficient gluconeogenesis (Ruan et al., 2012). This process is regulated by glucose availability, highlighting the important role of BAP1 in nutrient sensing. Additionally, liver-specific BAP1 knockout mice exhibit severe metabolic abnormalities, including impaired gluconeogenesis, hypoglycemia, and reduced hepatic glycogen stores, underscoring the critical function of BAP1 in glucose metabolism (Baughman et al., 2016). Lactate production in melanoma cells is also influenced by the interaction between BAP1 and LDHA. Studies have shown that BAP1, independently of its deubiquitinase activity, directly binds LDHA and sequesters it in the nucleus, thereby reducing cytoplasmic LDHA levels and suppressing lactate production. When BAP1 is inactivated, LDHA accumulates in the cytoplasm, resulting in enhanced glycolysis, substantial lactate production, and formation of an acidic tumor microenvironment (TME). Experimental evidence has shown that reintroduction of wild-type BAP1 reverses the lactate-elevation phenotype, whereas BAP1 mutants lacking a functional nuclear localization signal lose this capability (Zheng et al., 2024).

#### Lipid metabolism

BAP1 is also involved in the regulation of lipid metabolism. BAP1 deficiency leads to a marked reduction in hepatic lipid content, supporting an essential role for BAP1 in maintaining lipid homeostasis (Baughman et al., 2016). BAP1 has been shown to promote ACSL4 transcription by reducing H2Aub levels at the ACSL4 locus and enhancing chromatin accessibility. As a member of the long-chain fatty acyl-CoA synthetase family, ACSL4 catalyzes the activation of polyunsaturated fatty acids (PUFAs) into acyl-CoAs and serves as a major enzyme in lipid synthesis. Thus, BAP1 promotes ACSL4-driven lipid metabolic reprogramming through H2Aub-dependent epigenetic regulation (Fan et al., 2025). Furthermore, ASXL2, an important BAP1-interacting factor, has been reported to modulate both glucose and lipid homeostasis, suggesting that the ASXL2-BAP1 complex may act synergistically in metabolic regulation (Izawa et al., 2015). The tumor suppressor LKB1 is also stabilized by BAP1 through deubiquitination, thereby indirectly influencing AMPK pathway activity. As a key cellular energy sensor, AMPK regulates multiple aspects of glucose and lipid metabolism, thereby further expanding the BAP1 regulatory network in metabolic control (Yang et al., 2020).

### Cell death regulation by BAP1

**FIGURE 3:**
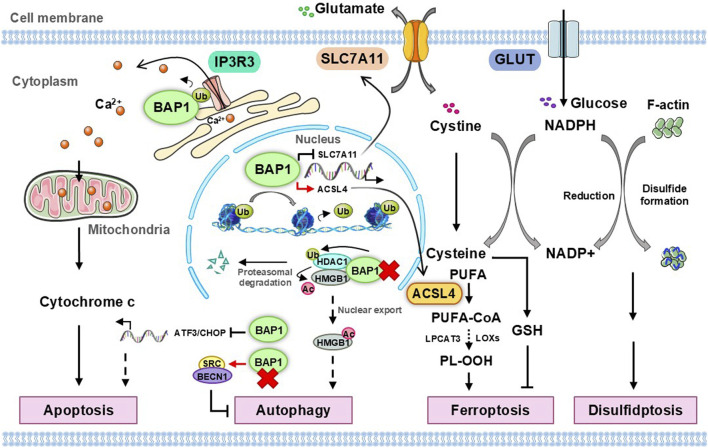
Summary of the roles of BAP1 in multiple forms of regulated cell death. BAP1 is involved in the regulation of apoptosis through mediators including IP3R3, ATF3, and CHOP. Autophagy is influenced by BAP1 through direct or indirect modulation of factors such as SRC and HMGB1. Ferroptosis is modulated by BAP1 through the SLC7A11-mediated cystine metabolism pathway and the ACSL4-dependent lipid metabolism pathway. BAP1 also influences disulfidptosis through processes linked to NADPH redox balance and intracellular disulfide formation, both of which are closely associated with SLC7A11 activity and glucose availability. This integrated model illustrates how BAP1, through its multifaceted regulatory functions, contributes to the regulation of cell fate among apoptosis, autophagy, ferroptosis, and disulfidptosis.

As a central regulator of cell death pathways, BAP1 has been reported to participate in metabolically linked cell death processes through diverse molecular mechanisms, including apoptosis, ferroptosis, and disulfidptosis (Figure 3).

#### Apoptosis

BAP1 localizes to the endoplasmic reticulum and activates the intrinsic apoptotic pathway either by binding to and deubiquitinating IP3R3, thereby promoting calcium release into the cytoplasm and mitochondria, or by interacting with 14-3-3 proteins to release the pro-apoptotic protein Bax (Bononi et al., 2017a; Sime et al., 2018). Inhibition of BAP1 reduces cellular sensitivity to calcium-dependent apoptosis, potentially providing a survival advantage for cells under stress. BAP1 has also been reported to promote cell survival under metabolic stress conditions, such as glucose starvation, by repressing the ER stress-related transcriptional regulatory network. BAP1 can bind to and repress the promoters of activating transcription factor 3 (ATF3) and C/EBP homologous protein (CHOP), thereby inhibiting unfolded protein response (UPR) activation, reducing ROS accumulation and ATP depletion, and ultimately attenuating apoptosis (Dai et al., 2017).

#### Ferroptosis

BAP1 has been identified as a key positive regulator of ferroptosis. Ferroptosis is promoted by BAP1 through deubiquitination of H2AK119ub at the SLC7A11 promoter, thereby repressing transcription of this critical cystine transporter. This repression reduces GSH synthesis, weakens cellular antioxidant defenses, and facilitates lipid peroxide accumulation (Zhang et al., 2018). ACSL4 is another transcriptional target positively regulated by BAP1 through epigenetic mechanisms; this regulation enhances ACSL4-mediated lipid metabolism and provides abundant substrates for ferroptosis-associated lipid peroxidation (Fan et al., 2025). BAP1 coordinates SLC7A11-mediated cystine metabolism and ACSL4-mediated lipid metabolism, thereby shaping tumor cell susceptibility to ferroptosis. Given the marked heterogeneity of tumors, these two pathways may represent complementary metabolic vulnerabilities, and additional BAP1-regulated mechanisms are likely to be identified. Together, these insights provide a conceptual framework and translational opportunities for developing metabolism-targeted therapies for BAP1-deficient tumors.

#### Disulfidptosis

Disulfidptosis, a form of programmed cell death, is primarily triggered by excessive intracellular disulfide accumulation. Under conditions of glucose deprivation or other oxidative stress, high SLC7A11 expression, which is observed in many cancer cells, can lead to aberrant accumulation of intracellular disulfide molecules, induce disulfide stress, and trigger disulfidptosis. This process is characterized by aberrant disulfide cross-linking of actin cytoskeletal proteins (Liu et al., 2023). By suppressing SLC7A11 expression, BAP1 prevents abnormal intracellular disulfide accumulation and cytoskeletal protein cross-linking that would otherwise occur as a result of excessive cystine uptake under NADPH-depleted conditions during glucose starvation. This mechanism may also explain the established role of BAP1 in promoting ferroptosis. Furthermore, cells with high BAP1 expression have been reported to exhibit a lower NADP+/NADPH ratio. Bioinformatics analysis of clear cell renal cell carcinoma patients showed a positive correlation between BAP1 expression and several NADPH metabolism-related genes, suggesting that BAP1 may enhance resistance to disulfidptosis by modulating the intracellular redox state (Wang et al., 2024). This protective role, which appears paradoxical relative to the canonical tumor-suppressive function of BAP1, may be context-dependent.

#### Autophagy

Recent studies have further elucidated the key role of BAP1 in autophagy regulation. As a tumor suppressor, BAP1 transcriptionally represses the expression of the proto-oncogene tyrosine-protein kinase SRC. When BAP1 is deficient, SRC levels are abnormally increased. SRC then binds to and phosphorylates specific tyrosine residues in the core autophagy protein BECN1, thereby inhibiting autophagy (Vega-Rubin-de-Celis et al., 2025). In normal cells, nuclear BAP1 deubiquitinates and stabilizes histone deacetylase 1 (HDAC1). HDAC1 subsequently deacetylates high-mobility group box one protein (HMGB1), and the deacetylated form of HMGB1 is retained in the nucleus. In contrast, in mesothelioma cells harboring mutant and nonfunctional BAP1, HDAC1 becomes unstable and undergoes degradation. This event leads to HMGB1 hyperacetylation, nuclear-to-cytoplasmic translocation, and cytoplasmic activation of autophagy. These findings directly link the tumor-suppressive function of BAP1 to HMGB1 subcellular localization and autophagic activity (Carbone et al., 2023).

### BAP1 and cancer

The functional inactivation of BAP1 represents a key molecular event that drives the initiation and progression of various tumors. BAP1 acts as a canonical tumor suppressor by regulating metabolic adaptation and cell death fate of tumor cells, thus modulating disease progression. These mechanisms provide an important biological basis for the development of targeted therapeutic strategies for BAP1-associated cancers.

### Clinical significance of BAP1 gene mutations in cancer

In 1971, Knudson proposed the seminal “two-hit hypothesis” in cancer biology, which posits that tumor initiation requires two mutational events that inactivate both alleles of the same gene. This model provided a foundational genetic explanation for carcinogenesis. BAP1 is a classic “two-hit” tumor suppressor gene, and its inactivation is frequently accompanied by loss of heterozygosity at the chromosome 3p21 locus or by biallelic deletion (Emi et al., 2015; Togo et al., 2016). In hereditary cancer syndromes, germline BAP1 mutations confer high susceptibility to multiple cancer types. The highest risk has been observed for malignant mesothelioma, followed by uveal melanoma, clear cell renal cell carcinoma, and cutaneous melanoma. These malignancies constitute the core spectrum of autosomal dominant BAP1-TPDS, which is caused by pathogenic germline BAP1 mutations (Walpole et al., 2018). Among carriers of germline BAP1 mutations, more than 85% are expected to develop one or more cancers during their lifetime, with penetrance approaching 100% with advancing age. Most patients with BAP1-TPDS have at least two close relatives affected by cancer (Abdel-Rahman et al., 2011; Rai et al., 2016; Pastorino et al., 2018; Walpole et al., 2018; Carbone et al., 2019; Carbone et al., 2022). Interestingly, a clinical paradox exists: germline BAP1 mutations are associated with a more favorable prognosis in mesothelioma, with a median survival of approximately 5–7 years compared with approximately 1 year in sporadic cases. In contrast, in uveal melanoma, germline BAP1 mutations are associated with poorer prognosis and increased metastatic risk. This paradox suggests that the function of BAP1 is highly dependent on cell type and TME.

**FIGURE 4:**
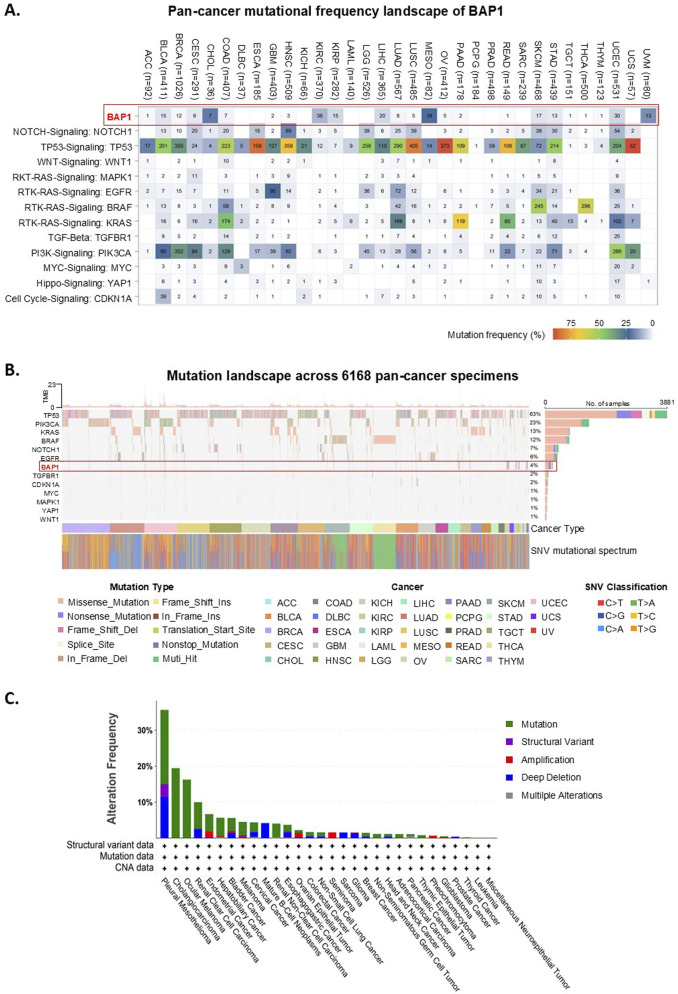
Pan-cancer mutational landscape of BAP1. **(A)** The horizontal axis represents different tumor types, whereas the vertical axis indicates BAP1 and multiple canonical oncogenic signaling pathways. The color scale denotes the proportion of patients with mutations among the total number of patients within each tumor type. A deeper red color indicates a higher mutation frequency, whereas white indicates no detected mutations or a mutation frequency of zero. The numbers within the colored blocks indicate the number of samples carrying mutations in the corresponding gene for each cancer type. “0” indicates that no mutation was detected in the coding region of the gene, whereas a blank block indicates that no mutation was detected in any region of the gene; **(B)** The waterfall plot shows the pan-cancer distribution of BAP1 mutations and the classification of specific SNV types. SNV data for 33 cancer types were obtained from the TCGA database. The TCGA SNV dataset included the following variant classifications: Missense_Mutation, Silent, 5′flanking region, 3′UTR, RNA, In_Frame_Del, Nonsense_Mutation, Splice_Site, Intron, 5′UTR, In_Frame_Ins, Frame_Shift_Del, Nonstop_Mutation, 3′flanking region, Frame_Shift_Ins and Translation_Start_Site. Silent, intronic, intergenic region (IGR), 3′UTR, 5′UTR, 3′flanking, and 5′flanking variants were excluded from the calculation of SNV percentages. The SNV mutation frequency in the coding region of each gene was calculated as the number of mutated samples divided by the total number of cancer samples. The oncoplot was generated using the maftools package; **(C)** Mutation frequencies of different BAP1 alteration types across cancer types. The horizontal axis represents different tumor types, and the vertical axis indicates the alteration frequency of BAP1. The height of each bar represents the alteration frequency, whereas different bar colors indicate distinct alteration types: green, mutation; purple, structural variant; red, amplification; blue, deep deletion; and gray, multiple alterations.

Somatic inactivation mutations in BAP1, which are highly prevalent across diverse sporadic cancers, have been consistently associated with poorer clinical outcomes in multiple cancer types (Carbone et al., 2020). Approximately 45% of primary uveal melanomas harbor BAP1 mutations, which are strongly associated with a poor-prognosis class 2 transcriptional signature and a metastatic phenotype (Harbour et al., 2010). The somatic mutation rate of BAP1 reaches 60%–70% in mesothelioma and approximately 15% in clear cell renal cell carcinoma (Peña-Llopis et al., 2012; Carbone et al., 2019). Lower frequencies have also been observed in thymic carcinoma, cholangiocarcinoma, and cutaneous melanoma (Togo et al., 2016). Analysis of The Cancer Genome Atlas (TCGA) database revealed that BAP1 is mutated in approximately 4% of pan-cancer cases, with missense mutations being the predominant alteration type (Figures 4A,B). Examination of single-nucleotide variant (SNV) data from 33 cancer types, together with cBioPortal profiling of distinct mutation classes, confirmed that BAP1 genomic alterations were enriched in pleural mesothelioma, uveal melanoma, and clear cell renal cell carcinoma. The major alteration types include inactivating point mutations, large-scale deep deletions, and structural variants, whereas gene amplification is rare across pan-cancer cohorts (Figures 4B,C). In general, BAP1 mutation is considered a critical indicator of adverse prognosis in uveal melanoma. BAP1 mutation may promote malignant transformation directly through the accumulation of genomic instability or by enhancing specific tumor susceptibility *via* gene-environment interactions (Novelli et al., 2021). By contrast, in cancers other than uveal melanoma, BAP1 mutations may often act as “passenger mutations” that do not directly drive tumor initiation, development, or progression. These mutations are frequently associated with high tumor mutational burden and UV exposure, show no significant impact on prognosis, and do not predict response to immunotherapy or targeted therapies (Matull et al., 2024).

### BAP1-mediated tumor suppression and its molecular mechanisms

In 2016, Kadariya et al. generated two knock-in mouse models mimicking human BAP1 cancer syndrome family mutations, along with one knockout model. Their experiments revealed that more than half of the BAP1-mutant mice spontaneously developed malignant tumors. Among these, two cases of spontaneous malignant mesothelioma were observed, and other common tumor types included ovarian sex cord-stromal tumors, lung cancer, and breast cancer. Further experiments with asbestos, an environmental carcinogen, demonstrated that BAP1 knock-in mice carrying clinically relevant mutations exhibited a significantly higher incidence of malignant mesothelioma than wild-type mice and had a shorter median survival. These findings revealed a synergistic effect of genetic predisposition and environmental factors in promoting cancer. Although the tumor spectra differ between humans and mice, with mice primarily developing ovarian tumors and humans more commonly presenting with mesothelioma and melanoma, both species exhibit tumorigenesis driven by a biallelic inactivation mechanism, and BAP1 mutation carriers show a significantly elevated risk of asbestos-induced carcinogenesis (Kadariya et al., 2016). These findings provide genetic evidence for the cross-species conservation of BAP1 tumor-suppressive function, confirming BAP1 as a *bona fide* tumor suppressor gene and offering crucial experimental evidence for insight into the pathogenesis of BAP1-TPDS.

BAP1 suppresses tumorigenesis *via* multiple mechanisms, including regulation of DNA damage repair, cell death, metabolism, and signal transduction pathways (Elsayed et al., 2025). Pancreas-specific BAP1 knockout mice have been observed to exhibit features of pancreatitis. In the context of a KrasG12D mutation, BAP1 knockout significantly accelerates pancreatic cancer progression and reduces mouse survival. Compared with wild-type pancreatic cancer cells, BAP1-knockout cells display genomic instability, defects in homologous recombination repair, and heightened sensitivity to radiotherapy and chemotherapy. Thus, BAP1 participates in DNA repair complex formation by directly binding BARD1 and influence gene expression by regulating histone modifications, thereby maintaining pancreatic homeostasis and suppressing tumorigenesis (Perkail et al., 2020). Zhao et al., using pancreatic cancer cell lines, a nude mouse model of pancreatic cancer lung metastasis, and KPC transgenic mice, demonstrated that BAP1 exerts tumor-suppressive function in pancreatic cancer in a catalytic activity-independent manner by inhibiting the IRAK1/4–NF-κB signaling pathway. IRAK1/4 inhibitors were found to effectively suppress BAP1-deficient pancreatic cancer (Zhao et al., 2025). Lee et al., using BAP1-deficient pancreatic cancer cells and pancreas-specific BAP1 knockout mice, demonstrated that BAP1 stabilizes LATS2, a key Hippo pathway inhibitor, thereby inhibiting downstream oncogenic effectors YAP/TAZ and exerting tumor-suppressive effects in pancreatic cancer (Brekken, 2020; Lee et al., 2020). In pancreatic ductal adenocarcinoma, genetic evidence further supports a causal role of BAP1 loss in tumor progression and immune resistance. Bap1 knockout in KPC mice increased tumor burden and shortened survival, and BAP1-deficient tumors failed to respond effectively to anti-PD-1 therapy. Mechanistically, BAP1 loss reduced CD8^+^ and CD4^+^ T-cell infiltration while increasing suppressive myeloid cell infiltration, indicating that BAP1 deficiency can remodel the tumor immune microenvironment and promote immune escape (Yuan et al., 2024).

Furthermore, BAP1 has been reported to inhibit cell migration and invasion and to enhance cisplatin-induced apoptosis in lung adenocarcinoma A549 cells. In a KRAS-mutant mouse model, the expression of both BAP1 and KEAP1 was progressively decreased during tumor progression and was accompanied by activation of the NRF2 pathway. Clinical data analysis showed that BAP1 mRNA levels were higher in normal lung tissue and that high BAP1 expression was significantly associated with improved survival among patients with lung adenocarcinoma, particularly those with advanced-stage disease. These findings further suggest that the tumor suppressive role of BAP1 in lung adenocarcinoma is mediated, at least in part, through regulation of the KEAP1-NRF2 pathway (Kang et al., 2022). Knocking down BAP1 in prostate cancer DU145 and P69 cells enhanced cancer cell migration, invasion, angiogenesis, and clonogenic growth, whereas BAP1 overexpression suppressed these malignant phenotypes. *In vivo* experiments further showed that BAP1 knockdown promoted mouse xenograft tumor growth, an effect that was completely reversed by PTEN reconstitution. Clinical data support a positive correlation between BAP1 and PTEN protein levels and an inverse correlation with Akt activation. Therefore, PTEN protein is directly stabilized by BAP1 through deubiquitination, thereby inhibiting the PI3K/Akt signaling pathway and mediating the tumor-suppressive effects of BAP1 (Deng et al., 2021).

In cholangiocarcinoma, valosin-containing protein (VCP) acts as a critical upstream regulator of BAP1. VCP directly binds to BAP1 and mediates its ubiquitination, thereby promoting BAP1 degradation through the ubiquitin-proteasome pathway, reducing BAP1 protein levels, and indirectly driving tumor progression. Blockade of ubiquitin-mediated degradation with the VCP inhibitor CB-5083 restored BAP1 protein expression, significantly inhibited cholangiocarcinoma growth, and induced apoptosis in both *in vitro* and *in vivo* models (Zhang et al., 2025). In hematologic malignancies, BAP1 deletion has been associated with approximately one-third of TP53-mutated acute myeloid leukemia (AML) cases. Using Bap1/Tp53 double-knockout mouse models combined with single-cell transcriptomic and epigenomic analyses, Andricovich et al. showed that BAP1 loss drives erythroleukemia through genomic instability and that aberrant H2AK119ub modification induces a shift in monocytic/granulocytic differentiation. These two parallel mechanisms cooperate with TP53 to promote leukemogenesis (Andricovich et al., 2025). Recent pan-cancer evidence further supports the functional consequences of BAP1 loss in tumor progression and DNA repair vulnerability. In BAP1-isogenic models of cholangiocarcinoma, mesothelioma, uveal melanoma, and clear cell renal cell carcinoma, reconstitution of wild-type BAP1 suppressed xenograft tumor growth, whereas BAP1 loss or expression of the catalytically inactive C91A mutant failed to do so, indicating that UCH-dependent deubiquitinase activity is required for BAP1-mediated tumor suppression. BAP1 was shown to support global genomic nucleotide excision repair by regulating the deubiquitination dynamics of the DNA damage recognition proteins damage-specific DNA binding protein 1 (DDB1), UV excision repair protein RAD23 homolog B (RAD23B), and COP9 signalosome subunit 7B (COPS7B). Loss of functional BAP1 impairs efficient DNA damage recognition and exposes BAP1-deficient tumors to synthetic vulnerabilities. Combined inhibition of lysine-specific demethylase 1 (LSD1) and PARP1 disrupts nucleotide excision repair, promotes apoptosis, and suppresses tumor growth in BAP1-deficient models (Hong et al., 2026).

Given the pivotal role of BAP1 in tumor suppression, further elucidation of the mechanisms and biological functions through which BAP1 regulates gene expression will provide deeper insight into its tumor-suppressive nature. This, in turn, will provide crucial theoretical basis and novel targets for the development of precision diagnostic and therapeutic strategies for BAP1-deficient or -mutated tumors.

### Oncogenic functions of BAP1 in regulating tumor cells

**FIGURE 5:**
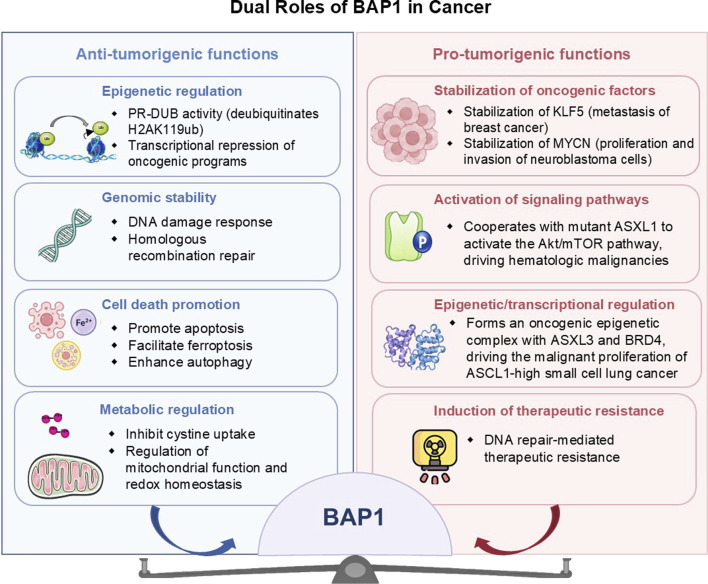
Dual functions of BAP1. BAP1 acts as a molecular hub that integrates multiple cellular processes and exhibits both tumor-suppressive and tumor-promoting activities depending on the biological context. In its well-established tumor suppressive role, BAP1 maintains genomic integrity and cellular homeostasis through epigenetic regulation, DNA damage response and homologous recombination repair, metabolic regulation, and promotion of cell death. In contrast, under specific genetic, epigenetic, cellular, or microenvironmental contexts, BAP1 may exert noncanonical tumor-promoting functions by stabilizing oncogenic proteins, modulating the TME, supporting metabolic reprogramming, and contributing to therapy resistance. The functional outcome of BAP1 activity is highly dependent on cell type, differentiation state, TME, and genetic/epigenetic context. TME: tumor microenvironment.

As research advances, the function of BAP1 has been found to exert context-dependence, indicating that its role is not limited to that of a traditional, singular tumor suppressor (Figure 5). BAP1 can form a complex with mutant ASXL1, directly deubiquitinating and stabilizing phosphorylated Akt, leading to sustained activation of the Akt/mTOR pathway. This process drives atypical proliferation and clonal expansion of hematopoietic stem cells while concurrently inducing mitochondrial dysfunction, ROS accumulation, and DNA damage, synergistically promoting the development of age-related clonal hematopoiesis and increasing the risk of malignant transformation (Fujino et al., 2021).

Qin et al. demonstrated that BAP1 promotes breast cancer cell proliferation, tumor growth, and lung metastasis by deubiquitinating and stabilizing the transcription factor KLF5, revealing a potential oncogenic role for BAP1 in breast cancer (Qin et al., 2015; Wang et al., 2025). In ASCL1-dependent small cell lung cancer (SCLC), BAP1 exerts an oncogenic function through its deubiquitinase activity. By stabilizing the scaffolding protein ASXL3, BAP1 forms an oncogenic BAP1/ASXL3/BRD4 epigenetic axis, which in turn activates ASCL1, a key transcription factor of the SCLC-A subtype, and its downstream proliferation- and survival-related target genes. The BAP1 inhibitor IBAP-II has also been shown to induce ASXL3 degradation, inhibit this key signaling axis, and significantly suppress SCLC cell viability and tumor growth in both *in vitro* and *in vivo* models (Tsuboyama et al., 2022).

BAP1 serves as a critical deubiquitinating enzyme for MYCN, a major oncogenic driver in neuroblastoma. Through direct interaction, BAP1 removes ubiquitin moieties from MYCN, thereby stabilizing its protein levels by inhibiting proteasomal turnover. This mechanism significantly maintains MYCN protein stability and ultimately enhances the proliferation and invasive capacity of neuroblastoma. As a consequence, BAP1 expression levels may serve as a potential prognostic biomarker, offering new therapeutic insights for high-risk, MYCN-amplified neuroblastoma (Zhang et al., 2023). Furthermore, Liu et al. found that BAP1, through its H2Aub deubiquitinase activity, promotes the recruitment and function of BRCA1, a key homologous recombination repair protein, at DNA damage sites. This process enhances the capacity of cancer cells to repair radiation-induced DNA damage, ultimately contributing to radiotherapy resistance (Liu et al., 2018).

**FIGURE 6:**
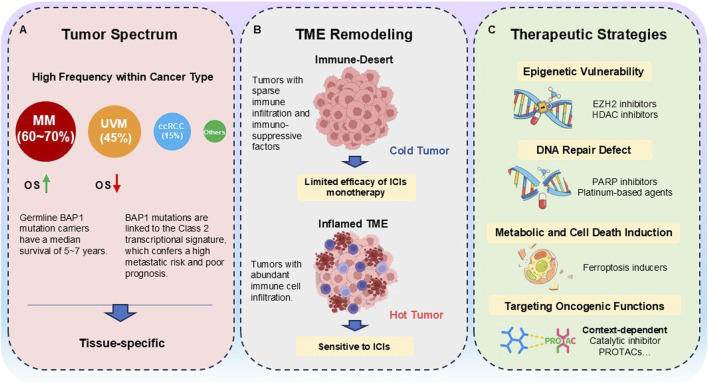
Clinical landscape and therapeutic vulnerabilities of BAP1-mutant cancers **(A)** Tumor spectrum and prognostic paradox: BAP1 mutation frequency and prognostic impact are tissue specific. Germline BAP1 mutations cause BAP1 tumor predisposition syndrome (BAP1-TPDS), which predominantly involves mesothelioma (MM), uveal melanoma (UVM), and clear cell renal cell carcinoma (ccRCC). Patients with germline BAP1-mutant mesothelioma exhibit longer survival than those with sporadic disease, whereas BAP1 mutations in UVM are associated with a class 2 signature, high metastatic risk, and poor prognosis, highlighting the context-dependent functions of BAP1; **(B)** TME remodeling: BAP1 mutations differentially reshape the immune microenvironment in a tissue-specific manner. Depending on the cellular context, this process may result in an inflamed, T-cell-infiltrated phenotype, referred to as a “hot tumor”, which may be sensitive to immune checkpoint inhibitors. Conversely, in other contexts, BAP1 loss may foster an immunosuppressive microenvironment, referred to as a “cold tumor”, characterized by sparse immune infiltration, thereby limiting the efficacy of immunotherapy alone; **(C)** Therapeutic roadmap: Targeting vulnerabilities arising from BAP1 loss provides four potential therapeutic strategies. Precision therapy requires the integration of tissue context, mutation status, and TME features. OS: overall survival; ICIs: immune checkpoint inhibitors; PROTAC: proteolysis-targeting chimera.

Taken together, these findings reveal the multifaceted clinical manifestations of BAP1-mutant tumors and their context-dependent therapeutic vulnerabilities (Figure 6).

### Therapeutic strategies targeting BAP1-mutant cancers

Although no clinical strategy currently exists to directly restore the function of inactivated BAP1, increasing research efforts have focused on exploiting the molecular vulnerabilities created by BAP1 loss or using BAP1 status as a biomarker to predict therapeutic sensitivity (Carbone et al., 2020). Current therapeutic approaches for BAP1-mutant cancers mainly target three interconnected biological features: epigenetic dysregulation, impaired DNA damage repair, and remodeling of the tumor immune microenvironment. In the epigenetic context, BAP1 loss is associated with aberrant chromatin regulation, increased H2A ubiquitination, and upregulation of epigenetic modifiers such as EZH2 and HDACs. Accordingly, EZH2 inhibitors, including tazemetostat, and HDAC inhibitors, such as vorinostat, panobinostat, and quisinostat, have shown antitumor activity in preclinical models of BAP1-deficient mesothelioma and uveal melanoma. However, their clinical efficacy remains variable, with EZH2 inhibition producing disease control in a subset of BAP1-inactivated mesothelioma patients but limited objective responses, and HDAC inhibitors showing inconsistent benefits in unselected clinical populations (Sun et al., 2018; Carbone et al., 2019; Kuznetsoff et al., 2021). In addition, some evidence has demonstrated that DNA methyltransferase (DNMT) inhibitors are effective in reshaping the characteristics of the TME, thereby improving antitumor immune responses (Yang et al., 2023; Zhong et al., 2023).

Given the role of BAP1 in homologous recombination-mediated DNA damage repair, platinum-based chemotherapy and PARP inhibitors have also been explored as rational therapeutic strategies. Several studies suggest that BAP1-mutant mesotheliomas, particularly pleural mesotheliomas, may exhibit increased sensitivity to platinum-pemetrexed chemotherapy, although conflicting clinical observations indicate that the predictive value of BAP1 alterations may depend on tumor site, mutation type, and coexisting DNA repair defects (Hassan et al., 2019). Similarly, PARP inhibitors such as olaparib, niraparib, and rucaparib have been investigated based on the concept of synthetic lethality. However, clinical responses have been inconsistent, potentially because BAP1-mutant cells may retain survival capacity despite homologous recombination impairment, partly due to intrinsic resistance to apoptosis (Ghafoor et al., 2021; George et al., 2024).

In addition, BAP1 mutations can profoundly influence the tumor immune microenvironment, making immunotherapy another important area of investigation. In mesothelioma, BAP1 loss has been associated with an inflammatory microenvironment, increased immune cell infiltration, and enhanced expression of immune checkpoint molecules, suggesting potential sensitivity to immune checkpoint blockade (Ladanyi et al., 2019; Shrestha et al., 2019). In contrast, BAP1-mutant uveal melanoma often exhibits an immunosuppressive milieu characterized by regulatory immune activation and upregulation of markers such as CD38 and CD74, which may partly explain the limited efficacy of immune checkpoint inhibitor monotherapy in this cancer type (Figueiredo et al., 2020). Similarly, in renal cell carcinoma, BAP1 mutation has been linked to CCR5-mediated immune suppression, raising the possibility that CCR5 blockade may serve as an adjunctive immunotherapeutic strategy (Zhou et al., 2020).

Overall, although BAP1 has not yet become a directly druggable target, its mutation status provides important therapeutic and predictive information. The available evidence indicates that the clinical relevance of BAP1 alterations is highly tissue-specific and context-dependent. Therefore, future therapeutic development should focus on biomarker-guided patient stratification and rational combination strategies. Such tailored approaches may better exploit the distinct molecular vulnerabilities of BAP1-mutant cancers and improve therapeutic outcomes.

## Concluding remarks

Beyond the established findings discussed above, understanding of the structure and function of the tumor suppressor gene BAP1 continues to expand as new evidence emerges. It is therefore likely that BAP1 research is entering a new phase that will involve increasingly complex mechanistic and translational challenges.

First, the molecular mechanisms that precisely regulate BAP1 structure and function, the cooperative oncogenic effects of BAP1 mutations, and the interactions between BAP1 alterations and environmental factors remain incompletely understood. A recent study demonstrated that BAP1, as the catalytic core of the PR-DUB complex, is allosterically regulated by monoubiquitination of ASXL1 at K351. This ubiquitination functions as a proteinaceous molecular glue that bridges and stabilizes the dynamic interface between BAP1 and the ASXL1 subunit. Without altering the binding affinity of PR-DUB for its nucleosomal substrate, this modification reduces conformational fluctuations and preserves the spatial integrity of key residues within the deubiquitinase catalytic pocket, thereby allosterically activating PR-DUB catalytic activity and markedly increasing its maximum reaction rate (Zhang et al., 2026). This finding reshapes current understanding of BAP1 functional regulation by showing that its activity depends not only on substrate recognition or complex assembly but also on precise post-translational control through conformational restraint. It also identifies a targetable allosteric regulatory site at the BAP1–ASXL1 interface, thereby opening new therapeutic strategies and design opportunities for allosteric inhibitors or pathway modulators targeting cancers associated with BAP1 loss or mutation. However, BAP1 itself contains multiple post-translational modification sites. How these modifications integrate upstream signals and dynamically regulate PR-DUB complex assembly and activity remains unclear. Moreover, several key questions require further investigation, including the identity of noncanonical BAP1 substrates, the molecular basis of BAP1 substrate selectivity, its potential functions in non-histone deubiquitination, and the impact of its chromatin-remodeling activity on transcriptional regulation. Second, tumors driven by BAP1 mutation exhibit marked tissue tropism. The physiological and pathological drivers underlying this specificity remain elusive. Initially, this tissue specificity was thought to be related to cell-selective evasion of apoptosis (He et al., 2019). Subsequent independent studies have shown that across different tissues, BAP1 loss disrupts specific downstream biological processes, including metabolic regulation, epigenetic plasticity, and hormonal responses, thereby conferring distinct oncogenic advantages (Waters et al., 2024; Zheng et al., 2024; Fan et al., 2025). This issue has substantial clinical relevance for explaining tumor distribution patterns, developing tissue-specific targeted therapies, and enabling precise early screening of high-risk populations. Third, because germline and somatic BAP1 mutations differentially affect patient prognosis and current treatments have limited efficacy in BAP1-mutant tumors, the development of effective and broadly applicable therapeutic agents and combination strategies has become increasingly urgent. For BAP1-mutant tumors that are insensitive to standard therapies, no approved treatments currently target the BAP1 protein itself directly. However, several indirect targeting strategies have been developed on the basis of BAP1-related biological functions. HDAC inhibitors may partially reverse the epigenetic imbalance caused by BAP1 loss (Landreville et al., 2012). EZH2 inhibitors can counteract EZH2 upregulation, which is associated with poor prognosis in BAP1-deficient tumors (LaFave et al., 2015; Sun et al., 2018). Given the DNA damage repair defects caused by BAP1 mutation, platinum-based chemotherapy or PARP inhibitors may be more effective in selected BAP1-mutant contexts (Louie and Kurzrock, 2020). Notably, preliminary clinical trial data indicate that biomarker-based patient stratification, including stratification by inactivating BAP1 mutations, is critical for improving the efficacy of these targeted approaches. More recently, the combined use of SRC inhibitors and autophagy inducers has been shown to exert significant synergistic antitumor effects in BAP1-mutant tumors, revealing a combination strategy that targets regulated cell death (Vega-Rubin-de-Celis et al., 2025). As understanding of the structure and function of BAP1 continues to advance, additional regulatory mechanisms and therapeutic vulnerabilities are expected to be identified, providing new directions for mechanism-based cancer treatment.
